# Clinical trials of self-replicating RNA-based cancer vaccines

**DOI:** 10.1038/s41417-023-00587-1

**Published:** 2023-02-10

**Authors:** Michael A. Morse, Erika J. Crosby, Jeremy Force, Takuya Osada, Amy C. Hobeika, Zachary C. Hartman, Peter Berglund, Jonathan Smith, H. Kim Lyerly

**Affiliations:** 1 Department of Medicine, Division of Medical Oncology, Duke University School of Medicine, Durham, NC USA; 2 Center for Applied Therapeutics, Department of Surgery, Duke University School of Medicine, Durham, NC USA; 3 HDT Bio, Seattle, WA USA; 4 VLP Therapeutics, Gaithersburg, MD USA

**Keywords:** Cancer, Drug development

## Abstract

Therapeutic cancer vaccines, designed to activate immune effectors against tumor antigens, utilize a number of different platforms for antigen delivery. Among these are messenger RNAs (mRNA), successfully deployed in some prophylactic SARS-CoV2 vaccines. To enhance the immunogenicity of mRNA-delivered epitopes, self-replicating RNAs (srRNA) that markedly increase epitope expression have been developed. These vectors are derived from positive-strand RNA viruses in which the structural protein genes have been replaced with heterologous genes of interest, and the structural proteins are provided in trans to create single cycle viral replicon particles (VRPs). Clinical stage srRNA vectors have been derived from alphaviruses, including Venezuelan Equine Encephalitis (VEE), Sindbis, and Semliki Forest virus (SFV) and have encoded the tumor antigens carcinoembryonic antigen (CEA), human epidermal growth factor receptor 2 (HER2), prostate specific membrane antigen (PSMA), and human papilloma virus (HPV) antigens E6 and E7. Adverse events have mainly been grade 1 toxicities and minimal injection site reactions. We review here the clinical experience with these vaccines and our recent safety data from a study combining a VRP encoding HER2 plus an anti-PD1 monoclonal antibody (pembrolizumab). This experience with VRP-based srRNA supports recent development of fully synthetic srRNA technologies, where the viral structural proteins are replaced with protective lipid nanoparticles (LNP), cationic nanoemulsions or polymers.

## Introduction

Numerous platforms (proteins, peptides, viral vectors, dendritic cells, exosomes, DNA plasmids, and RNA) have been adapted for use as therapeutic cancer vaccines in an attempt to activate T cell and antibody responses against tumor-expressed antigens. Although there has been longstanding enthusiasm for using mRNA as a vaccine platform [1], based on its safety (inability to integrate into the genome or generate infectious virus), innate immunity induction [2], efficient manufacture, and convenience, challenges have included its short half-life and transient protein expression. As described elsewhere in this series and reviewed by others [3], self-replicating, single-stranded, positive sense RNA (srRNA), encoding replicases, and target tumor antigens in place of viral structural proteins, generate large amounts of mRNA coding for the target tumor antigen, and they can deliver a larger amount of antigen over a longer period of time than nonreplicating mRNA. Preclinical and clinical data have demonstrated potent and durable immune responses, rapid production, ease of administration, and safety. The favorable clinical experience with srRNA for infectious disease applications, substantially further along in development, has provided support for extension of these vaccines into cancer treatment where immunogenicity and the safety and feasibility of combination with other therapies can be complex. The clinical experience with srRNA vectors in patients with malignancy has mainly consisted of studies with viral replicon particles (VRP) derived from the alphaviruses Venezuelan Equine Encephalitis virus (VEE), Sindbis, and Semliki Forest Virus (SFV). Nonetheless, early studies with completely synthetic srRNAs where the viral structural proteins are replaced with a protective coat in the form of a lipid nanoparticle (LNP) have also demonstrated safety and immunogenicity. We will review this prior experience (see Table 1 for summarized data) and, because combinatorial strategies are the next stage in development for the cancer immunotherapy field, we will provide preliminary clinical data from the initial safety phase of a phase II clinical trial testing a combination of a self-replicating RNA and the anti-PD-1 antibody pembrolizumab in advanced HER2 + breast cancer patients.

**Table 1:** Published and presented clinical trials of self-replicating RNA cancer vaccines.

Ref NCT # phase name of vaccine	Based on RNA virus	VRP or LNP	Antigen	Doses administered	Number of patients treated	Toxicities	Tumor antigen specific immune response	Clinical response/outcomes
6 NCT00529984 Phase I VRP-CEA(6D)	VEE	VRP	CEA	4 × 10^7^ IU per 0.5 ml and 4 × 10^8^ IU per 0.5 mL IM. Doses every 3 weeks for 4 immunizations. Additional boosters allowed if stable.	28 M:18, F:10. CRC: 23 NSCLC: 2 Panc: 1 Append: 1 Breast: 1	No grade 3,4 toxicity attributed to vaccine. 2 with grade 1 injection site pain	T and B cell	1 CR, 2 SD 2 with NED remained without progression. Better survival with an immune response by ELISpot
9 NCT # not listed Phase I PSMA-VRP	VEE	VRP	PSMA	1.8 × 10^7^ and 0.72 × 10^8^ IU/mL SC. Doses at weeks 1, 4, 7, 10, and 18	12 M:12 Metastatic CRPC: 12	No grade 3,4 adverse events attributable to vaccine; Gr 1/2 fatigue in 8 patients	B cell	Two patients from each dose level showed marked reduction in the N-telopeptide levels (marker of bone turnover)
10 NCT01526473 Phase I VRP-HER2	VEE	VRP	HER2	5.2 × 10^8^ IU/mL IM. Doses every 2 weeks for 3 doses.	22 M:1 F: 21 Median age 58.5 (range 53–64) Breast: 21 Esoph: 1	No grade 3,4 adverse reactions 2 Grade 1 injection site reactions	T and B cell	1 PR/2 SD. mOS 50.2 mo in cohort 1; 32.7 mo in cohort 2. Perforin expression by memory CD8 T cells post-vaccination significantly correlated with improved PFS.
7 NCT01890213 Pilot VRP-CEA(6D)	VEE	VRP	CEA	4 × 10^8^ IU per 0.5 mL IM Doses every 3 weeks × 4 doses	12 M:4 F:8 Median age 53 (IQR 43–62) All CRC stage III	No grade 3,4 adverse events. 3 grade 1 injection site reactions, 2 grade 1 fever	T and B cell	5-year RFS was 75%, (95%CI 40–91%);
11 NCT # not listed Phase I Vvax001	SFV	VRP	HPV E6, E7	1.25 × 10^8^ IP/mL IM Doses every 3 weeks × 3 doses. Each dose was given as two injections, one IM in 1 mL in each upper leg	12 F: 12 HPV-induced CIN 2 or 3	Mild injection site reaction, injection site hematoma, peripheral edema, chills, myalgia, back pain, and lymphadenopathy	T cell	NR
16 NCT03639714 Phase I GRT-C901/ GRT-R902	Prime: ChAd Boost: self amplify-ing RNA	LNP	Individualized neoepitopes	IM Dose not listed + 30 mg SC ipilimumab and 480 mg IV nivolumab	26 (18 evaluable) (NSCLC, CRC, gastro-esoph, urothelial)	Injection site reactions and fever	T cell (and B cell)	1 CR, 4 SD, 11 PD, and 2 no measurable disease. 4/5 had decrease in ctDNA
17 NCT03953235 Phase I GRT-C903/ GRT-R904	Prime: ChAd Boost: self amplify-ing RNA	LNP	Shared neoepitopes (of KRAS, NRAS, BRAF and others)	IM Dose not listed + 30 mg SC ipilimumab and 480 mg IV nivolumab	26 (NSCLC, CRC, pancreatic cancer, and others with shared epitopes)	Injection site reactions and fever	Not reported	66% of NSCLC patients had decrease in ctDNA

*ChAd* Chimpanzee Adenovirus, *SFV* Semliki Forest Virus, *VEE* Venezuelan Equine Encephalitis, *IP* infectious particles, *SC* subcutaneous, *IV* intravenous, *IM* intramuscular, *CRC* colorectal, *NSCLC* non-small cell lung cancer, *CRPC* castrate resistant prostate cancer, *CIN* cervical intraepithelial neoplasia.

## VEE VRP expressing enhanced carcinoembryonic antigen (CEA(6D)): (VRP-CEA(6D), AVX-701)

### Vaccine description

Because carcinoembryonic antigen (CEA) is widely expressed in many adenocarcinomas, it has been a frequently targeted molecule in immunotherapy strategies [4]. VRP-CEA(6D) (AVX-701) is a VEE-based VRP vaccine, which expresses CEA(6D), a modified version of CEA with an Asn to Asp substitution in the HLA-A2 binding motif, which enhances recognition by T cell receptors [5].

### First in human clinical study design

Based on preclinical safety and immunogenicity [6], we performed a standard 3 + 3 dose escalation/expansion phase I clinical trial of patients (*n* = 28) with heavily pre-treated metastatic cancer expressing CEA (predominantly colorectal) to establish safety, immunogenicity, and a recommended dose for further study [6] (NCT00529984). The VRP-CEA(6D) injections were administered intramuscularly (alternating sides of the body) to cohorts of 3–6 patients at doses of 4 × 10^7^ IU, 1 × 10^8^ IU, or 4 × 10^8^ IU every 3 weeks for 4 immunizations. These doses were ultimately limited by the amount of clinical-grade study drug generated for the study. When the dose of 4 × 10^8^ IU was found to be without dose limiting toxicity, an additional cohort of 14 subjects was enrolled at this dose to obtain more data on immunogenicity. Of these patients, 10 completed the 4 immunizations, while 4 received fewer due to progression of disease. Booster doses every 3 months (until progression of disease) were received by 2 patients with stable disease after the initial four doses.

### Tolerability and toxicity

The VRP-CEA(6D) was well-tolerated with no grade 4 events and the six grade 3 events were all attributed to disease progression. There were minimal injection site reactions (two patients had grade 1 injection site pain) and no fevers reported.

### Immune responses

CEA-specific immune responses, assessed by anti-CEA antibody titer and T cell assays (enzyme-linked immunosorbent spot (ELISPOT) and cytokine flow cytometry) increased after immunization beginning with the second or third dose and appeared to plateau after the initial four doses. At 3 months after the 4th dose, CEA-specific immune responses remained elevated (though longer-term immunogenicity data was not collected). Both CD4 + and CD8 + CEA-specific T cell responses were detected. The CEA-specific antibody sera were able to bind to CEA expressing tumor cells and had ADCC activity.

Because VEE is not endemic in the patient population studied, no patients had preexisting VEE specific antibodies. Consequently, in addition to the CEA-specific immune response, the anti-VEE antibody titer also markedly increased post vaccination and remained elevated throughout the immunizations, as expected. Although these antibodies would be neutralizing to natural VEE infection, there was no correlation between the neutralizing titer and anti-CEA antibody response suggesting that the VRP remained functional despite the presence of anti-VEE antibodies.

As previously reported for advanced cancer patients, CD4posCD25hiFoxP3pos regulatory T cell (Treg) levels in our study participants were higher than those of healthy controls; however, the immunizations did not increase Treg and the magnitude of the CEA-specific T cell and antibody responses did not differ between peripheral blood Treg levels demonstrating that the VRP-CEA(6D) immunizations could break tolerance to CEA regardless of the immunosuppressive effects of Treg.

### Clinical responses

Of the 28 patients enrolled, all refractory to prior standard therapies, there was a patient who experienced a complete response of a small liver lesion and two patients showed stable disease. Two others who had no evidence of disease prior to immunization continued with no recurrence. There was a trend for improved survival in the patients with an ELISpot-detected response.

### Long-term follow up

We subsequently performed a follow-up analysis of surviving stage IV colorectal cancer patients who had participated in the phase I study [7]. Of the original 28 patients enrolled, three were alive at 9.6, 10.5, and 11.4 years, respectively, all of whom had been rendered free of metastatic disease by prior interventions disease (but were predicted to have a high risk of recurrence) at the time they enrolled in the study of VRP-CEA(6D). This suggested that patients with the lowest tumor burden may have greater benefit from the vaccinations, which was tested in a study conducted in patients following surgical resection of their tumors.

### Post-surgical resection clinical study design

Based on our initial study and the hypothesis that immune responses would be superior in patients with a lower tumor burden, we initiated a follow up pilot study of the VRP-CEA(6D) in patients with resected stage III colorectal cancer who had completed standard adjuvant chemotherapy (fluoropyrimidine with or without oxaliplatin) but had no evidence of disease, yet an increased risk of recurrence [7]. The goal of the study was to demonstrate immunogenicity of the vaccine in less heavily pre-treated patients. VRP-CEA(6D) (4 × 10^8^ IU) was injected intramuscularly (alternating arms) every 3 weeks for four doses. Patients were followed at six-month intervals with imaging studies to evaluate for recurrence of disease.

### Tolerability and toxicity

There were grade 3 or 4 toxicities attributed to the injections and all twelve patients completed the immunizations. Three had grade 1 injection site reactions, 2 had grade 1 injection site pain, 2 had grade 1 fever, and 1 had grade 1 flu like symptoms.

### Immune responses

For consistency with our previous immune analysis strategy, we performed CEA-specific IFNγ-ELISPOT, and observed an increase in the frequency of circulating T cells secreting IFNγ induced by the immunizations in the patients initially tested. The subsequent introduction of higher dimensional multiparameter cytometric methods utilizing mass cytometry (CyTOF) permitted a more comprehensive picture of the immune response to the VRP-CEA(6D) immunizations. Important observations were increases in CD8 + granzyme B + central memory T cells capable of secreting IFNγ in 9/12 (75%) and CD8 + TEM (and more specifically, the terminally differentiated effector memory cells (CD8 TEMRA) in 10/12 (83.3%) patients. Consistent with prior observations, Tregs did not increase and in fact, decreased in 10/12 (83.3%) patients following the immunizations, resulting in an increase in the CD8 TEMRA:Treg ratio in 10/12 (83.3%) patients. Anti-CEA antibodies were also activated by the immunizations in all patients; however, the titer was higher than previously observed in the stage IV populations suggesting better preserved immune responsiveness in these patients with no evidence of active malignancy. Importantly, all responses were observed despite the expected development of VRP-neutralizing antibodies. Taken together, these data confirmed that the CEA(6D) VRP could activate anti-tumor responses without inducing counter-regulatory, immunosuppressive responses and despite the induction of VEE-neutralizing antibodies.

### Clinical responses

After a median follow-up of 60 months, all patients remained alive and only 3/12 (25%) had experienced recurrent disease, which compares favorably with historical data for this mainly stage IIIB population of patients.

## VEE-based VRP expressing prostate-specific membrane antigen (PSMA): (PSMA-VRP)

### Vaccine description

A VEE-based VRP expressing prostate-specific membrane antigen (PSMA).

### Clinical study design

Building upon the preclinical efficacy of VEE-based VRPs expressing the ubiquitous prostate cancer antigen PSMA [8], a phase I first-in-human clinical trial was performed in patients with progressive castrate resistant prostate cancer (CRPC) metastatic to bone to establish safety and immunogenicity of the vaccine [9]. The PSMA-VRP, formulated at concentrations of 1.8 × 10^7^ and 0.72 × 10^8^ IU/mL, was administered as up to 5 injections subcutaneously in the subject’s deltoid region, alternating arms on weeks 1, 4, 7, 10, and 18. Twelve patients (average age 68.8 ± 10.9 years, the majority of whom had prior hormonal therapy and one with prior chemotherapy) were evaluable. Baseline characteristics were similar in all cohorts. The first 3 patients received 0.9 × 10^7^ IU, the next 3 received 0.36 × 10^7^ IU as did 6 patients in the expansion cohort. In the first cohort, 2 of 3 patients received the 5 doses and 6 of 9 who received the higher dose received all 5 vaccines. Progressive disease was the predominant reason for discontinuation.

### Tolerability and toxicity

The vaccine was well-tolerated with grade I/II events that were possibly related to study drug consisting of fatigue, anorexia, and weight loss. Two subjects who received 0.36 × 108 IU, had grade 3 events related to progressive prostate cancer. There were no vaccine-related grade III or higher events.

### Immune responses

Although patients’ PBMCs had appropriate mitogenic responses to stimulation by phytohemagglutinin (PHA) and CEF (T cell epitopes from Cytomegalovirus, Epstein-Barr virus, and Influenza virus peptides), there were no significant cellular responses detected against PSMA peptides (consisting of 15-mer peptides that overlapped by 10 amino acids spanning the sequence of PSMA). It was hypothesized that the doses chosen may have been too low to induce T cell-mediated immune response. Two patients had antibodies against PSMA expressing cells (3T3-PSMA) detectable by flow cytometry, both at the higher dose of the vaccine. Four patients, different from those with detectable PSMA antibodies, had a PSMA ELISA endpoint titer that exceeded 100, the majority in the higher dose cohort.

As seen in previous studies of VEE-based VRP, VEE neutralizing titers were commonly seen post-vaccination. The mean maximal neutralizing titer was 4–5 fold higher for subjects who received the higher dose and 5 doses. Despite the neutralizing titers (mean 1/9970), all patients who had neutralizing titers also had humoral responses to PSMA. This suggests that the neutralizing antibody does not affect the ability of VRP-PSMA to induce an immune response.

### Clinical responses

There were no significant clinical changes (in the tumor marker PSA or circulating tumor cells), although a decrease in markers of bone turnover was noted in some patients.

## VEE VRP expressing human epidermal growth factor receptor 2 (HER2): (VRP-HER2, AVX-901)

The safety profile of srRNA, in part due to the lack of a potential for genomic integration or cell transformation among other safety features incorporated into the design, allowed us to develop VEE-based VRP encoding genes considered oncogenic to human such as HER2.

### Vaccine description

VRP-HER2 is based on the same VEE-particle platform used for VRP-CEA(6D) (AVX-701), but with the extracellular (ECD) and transmembrane (TM) domains of the human HER2 gene replacing the structural protein genes [10]. HER2 is a well-established oncologic target for monoclonal antibodies, antibody-drug conjugates, and tyrosine kinase inhibitors, but others and we have previously demonstrated that vaccines encoding various domains of HER could also induce anti-HER2 T cell and antibody responses. As HER2 is oncogenic, functional deletions of the molecule were incorporated into the vaccine to further minimize any risk of transformation of cells transfected by the VRP vector, despite the lack of integration or chronic expression of HER2 in any transfected cells.

### Clinical study design

Based on favorable preclinical immunogenicity and antitumor profiles, the VRP-HER2 vector was advanced to a phase I clinical trial [10] for patients with metastatic or recurrent HER2 + malignancies (primarily breast cancer) defined as HER2 3+ by IHC or HER2 2+ by IHC with FISH amplification) who had progression of disease after prior HER2-targeted therapy (including trastuzumab, trastuzumab plus pertuzumab, T-DM1, or lapatinib). The goal was to establish safety and immunogenicity of the vaccine alone or in combination with other HER2 targeted therapies. All study participants received VRP-HER2 at 4 × 10^8^ IU intramuscularly, alternating arms, every 2 weeks for three administrations. The first cohort received VRP-HER2 monotherapy and the second cohort received the vaccine in combination with other HER2 targeted therapies, as this was reasoned to be the likely utilization of the vaccine in future studies.

### Tolerability and toxicity

The VRP-HER2 vaccinations were well tolerated, without dose limiting toxicity, and there were no grade 3 or 4 toxicities related to the immunizations. Adverse events possibly related to the VRP-HER2 included grade 2 fatigue and decreased white blood cell count and grade 1 diarrhea, rash, oral mucositis, malaise, nausea, dry mouth, sore throat, decreased neutrophil count, and decreased white blood cell count. Although clinical trials with HER2 targeted antibodies such as trastuzumab have reported events of cardiomyopathy, we did not observe any decreases in cardiac ejection fraction.

### Immune responses

CyTOF analysis demonstrated that an activated memory T cell population had increased perforin expression in response to HER2 peptides following immunization. Among those who experienced an increase in perforin expression by memory CD8 T cells following vaccination, PFS was longer than among those who did not. Despite development of neutralizing antibodies against VRP, anti-HER2 antibody titers increased in the majority of patients following the immunization. Although the second cohort of patients was receiving, or had recently received, trastuzumab with or without pertuzumab, we were able to detect vaccine induced HER2 antibodies in 14/17 participants by binding of the polyclonal sera to a cell line transfected with a form of human HER2 that was mutated so that it could not bind trastuzumab or pertuzumab. Further, the polyclonal sera could mediate a greater degree of ADCC and HER2 internalization following immunization.

### Clinical responses

In our first cohort, there were no responses and the median PFS was 1.8 months and the median OS was 50.2 months. A second cohort allowed the vaccine to be administered in combination with HER2 targeted therapy such as trastuzumab, despite previous progression while on HER2 targeted therapy. In this cohort, there was one partial response (PR) and seven with initially stable disease (SD), two of whom had continued SD. The median PFS for cohort 2 was 3.6 months and the median OS was 32.7 months. Among the subgroup of patients in cohort 2 who demonstrated an increase in perforin expression by memory CDS T cells following vaccination, there was a significantly longer PFS than among those who did not. These data demonstrate that the induction of a HER2-specific memory CD8 T-cell population following vaccination may represent a biomarker for immune responsiveness and beneficial clinical outcomes. Further, although Treg were observed to increase, remain stable or decrease across study participants, among patients in whom peripheral blood Tregs decreased, improved OS was observed, suggesting that Treg decrease may also be a potential biomarker for vaccine activity that can be further validated in larger studies.

## SFV based VRP encoding Human Papilloma Virus E6 and E7- (Vvax001)

### Vaccine description

Vvax001 is a replication-deficient recombinant Semliki Forest virus replicon particle encoding a fusion protein of the HPV16 antigens E6 and E7 [11].

### Clinical study design

Vvax001 was tested [11] in participants with surgically treated cervical intraepithelial neoplasia, a condition in which constitutive expression of the E6 and E7 proteins induce neoplastic transformation. Using a four cohort, 3 + 3 dose escalation design, Vvax001 at doses levels of 5 × 10^5^, 5 × 10^6^, 5 × 10^7^, and 2.5 × 10^8^ infectious particles (IP) per immunization (divided into 2 intramuscular injections, each given into the upper legs) was administered every 3 weeks for 3 immunizations. Goals of the study were to establish vaccine safety and immunogenicity, and a dose recommendation for future studies.

### Tolerability and toxicity

All dose levels were well-tolerated with no CTC grade 3,4 or dose-limiting toxicity and the treatment-emergent adverse events (TEAEs) related to study treatment were mild injection site reaction, injection site hematoma, peripheral edema, chills, myalgia, back pain, and lymphadenopathy. Only one injection site reaction was graded as moderate.

### Immune responses

ELISPOT analysis of peripheral blood mononuclear cells (PBMC) demonstrated immune responses against E6 and E7 peptide pools (E6 responses generally greater than E7 responses) at all dose levels with 5/12 participants responding following 2 immunizations and 10/12 after the three immunizations. While the highest frequency of E6/E7-specific T cells was observed at the highest dose level in one participant, there was also a participant at this level with no response (although their control samples had high responses which may have limited the ability to detect a response). Both CD4 + and CD8 + T cells responded to the peptides. A non-specific increase in T cell responsiveness was noted after the immunizations in the control samples not exposed to peptides. Proliferation assays detected dose-dependent proliferation of CD4 + T cells (greater than CD8 + T cells) after the immunizations and analysis of supernatants from the proliferation cultures demonstrated increased production of Th1 cytokines (IFNγ and CXCL10) but not type 2 cytokines (IL-4 and IL-10). Anti-vector antibodies were detected in participants after vaccination in all but the lowest dose cohort. Although the antibodies could neutralize SFV infection in vitro, their presence did not prevent higher levels of immune response after the repeated immunizations, consistent with the observations made in studies with VRP-CEA(6D).

### Clinical responses

No data regarding clinical outcomes (such as rates of development of cervical cancer) were reported in this study with short term follow-up. Further, no long-term safety data were reported.

## Sindbis virus based VRP expressing NY-ESO-1 (CYN102)

CYN102 (mentioned at https://www.sbir.gov/sbirsearch/detail/1910949) is based on Sindbis virus (SINV) and encodes the tumor antigen NY-ESO-1. This website indicates that Cynvec was to begin a Phase 1 clinical trial of CYN102 in women with chemotherapy-resistant EOC to establish its clinical safety; however, there are no studies listed in clinicaltrials.gov and no clinical trial reports are available for this construct.

## Combination srRNA cancer vaccine with checkpoint blockade

**Fig. 1:**
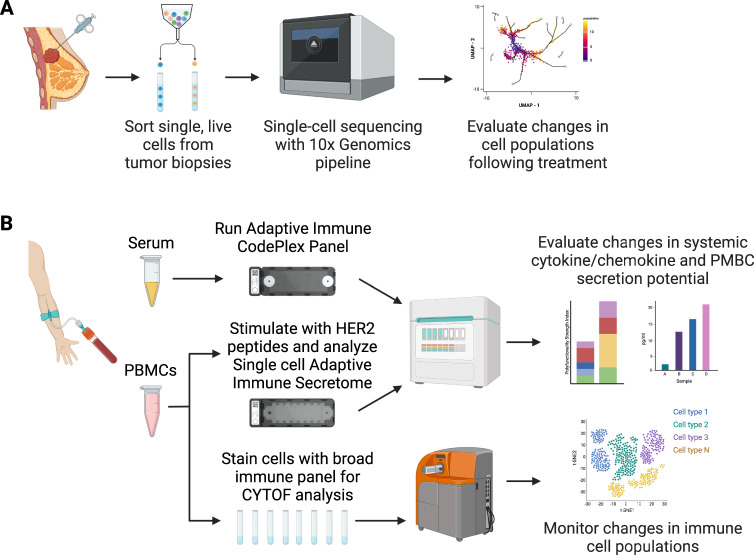
Schematic for immune analysis of biopsies and blood samples from safety lead-in cohort form pembrolizumab plus VRP-HER2 trial. **A** Biopsies pre- and post-vaccination were digested and single live cells were sorted and were then subjected to single-cell RNA sequencing. **B** Serum was analyzed for alterations of systemic cytokines. PBMCs were stimulated by an overlapping panel of HER2 peptides and functional cytokine/marker alterations of single cells were assessed by single cell secretome ELISA and Cytometry Time of Flight (CYTOF).

**Fig. 2:**
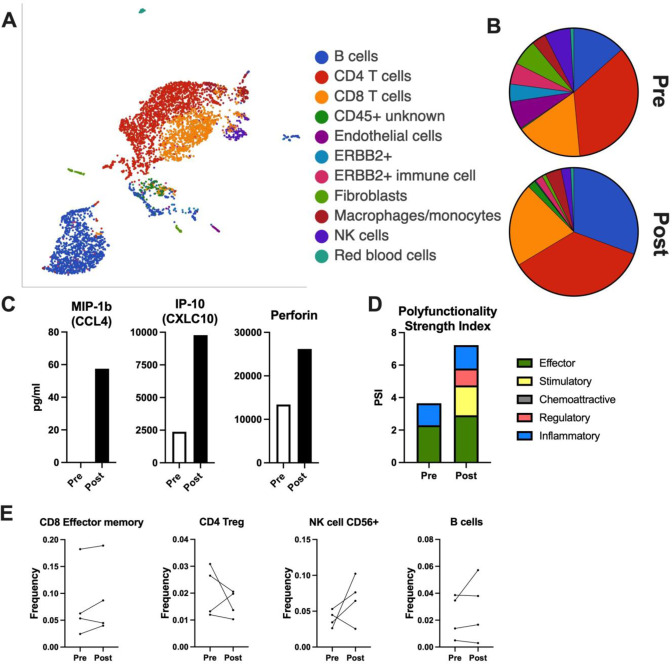
Interim immune analysis of patients treated with HER2-VRP and pembrolizumab. **A** Tumor biopsies were processed fresh for single-cell RNA sequencing of live cells using the 10x Genomics platform. Pre-treatment biopsies from 2 patients and post-treatment biopsies from 3 patients were pooled for analysis of relevant cell populations. **B** Summary of cellular composition in pre- and post-treatment biopsies. **C** Serum from patients pre- and post-treatment was analyzed for 22 cytokines using the CodePlex Adaptive Immune chip from Isoplexis. Top hits from a single patient are shown. **D** Peripheral blood mononuclear cells (PBMCs) were restimulated with pooled HER2 peptides and analyzed for the production of 32 cytokines and chemokines using the Adaptive Immune Single-Cell Secretome chip from Isoplexis. The Polyfunctionality Strength Index for the same patient for **C** is shown. **E** PBMCs from patients pre- and post-treatment were stained and analyzed for changes in circulating immune cells by CYTOF. Frequency of select populations from the first 4 patients is shown.

Based on preclinical data demonstrating enhanced anti-tumor activity for VRP-HER2 combined with anti-PD-1 antibodies [12], we have now initiated a randomized phase II clinical trial of VRP-HER2 versus pembrolizumab versus VRP-HER2 plus pembrolizumab in women with advanced HER2 + breast cancer who have received first line chemotherapy plus trastuzumab and pertuzumab and are continuing maintenance trastuzumab plus pembrolizumab (NCT03632941). The primary objective is to determine whether pembrolizumab increases tumor infiltrating T cells and HER2 specific antibodies induced by the VRP-HER2 vaccine. This protocol was approved by the Duke University Medical Center Institutional Review Board and participants gave written informed consent before taking part. This protocol requires enrollment of an initial cohort of 3 patients who receive pembrolizumab plus VRP-HER2 as a means to demonstrate overall safety prior to the randomized portion of the study. The VRP-HER2 (4 × 10^8^ IU intramuscularly) is given every 2 weeks for 3 doses and the pembrolizumab (200 mg IV) is given every 3 weeks for 5 doses starting on the day of the first VRP-HER2 injection. In these 3 patients, the immunizations plus pembrolizumab were well tolerated with no DLT. Tumor biopsies were performed before the initial immunization and eight weeks after the third VRP-HER2 immunization to assess alterations in the local tumor microenvironment. To assess cellular alteration, tumor biopsies were digested and live single cells obtained by flow cytometry. These cells were then subjected to single-cell RNA sequencing as described in Fig. 1A. To assess systemic immune responses pre- and post-vaccination, we also obtained serum and PBMCs from patients. Serum was assessed for alterations of systemic cytokines, while PBMCs were stimulated by an overlapping panel of HER2 peptides and functional cytokine/marker alterations of single cells assessed by single cell secretome ELISA and Cytometry Time of Flight (CYTOF) as detailed in Fig. 1B. Using these immune profiling techniques, we found within these patients an enhancement of lymphocyte infiltration into tumors post-vaccination, with augmented infiltration of both CD4 + and CD8 + T cells, as well as B cells (Fig. 2A-B). Single-cell analysis also revealed that 90% of HER2 transcripts post-vaccination are in immune cells, compared to 55% in pre-vaccination settings (Fig. 2A, B). This is congruent with an enhanced antibody-dependent cellular phagocytosis (ADCP) of HER2 + cells by the myeloid cell population after vaccination, compared to uptake mediated by HER2 mAbs alone [13]. This could be due to the induction of HER2-specific antibodies by the VRP-HER2 vaccine that we have previously documented in our Phase I trial [10], as well as enhanced ADCP mediated by PD-1 blockade [14, 15].

Analysis of systemic immunity likewise revealed the VRP-HER2 vaccination plus pembrolizumab to enhance immunity in patients with metastatic breast cancer. Specifically, analysis of PBMCs revealed an enhancement of chemokines and CD8 activation molecules (CXCL10, CCL4, and perforin) in serum post-treatment (Fig. 2C). Stimulation of PBMCs using HER2-specific peptides demonstrated that this vaccine plus PD1 blockade treatment enhanced the polyfunctionality index of HER2-specific T cells (Fig. 2D), augmenting the stimulatory and effector populations. Additionally, CYTOF analysis revealed an enhancement of CD8 effector memory T cells, NK cells, and B cells in some patients as well as a decrease in Treg populations (Fig. 2E). This is consistent with the induction of HER2-specific immunity elicited by vaccination, as well as the enhanced functional ability of these cells potentially mediated by PD-1 blockade. While these results are preliminary, they suggest the clinical potential of this approach, even in patients with advanced metastatic cancers.

## Fully synthetic self-replicating RNA expressing personalized cancer neoepitopes

As noted above, neutralizing antibodies against VEE-based VRP have frequently developed following vaccination. Although these antibodies did not seem to have an adverse impact on the immune response to the target antigen in these early studies, next generation, fully synthetic srRNA encapsulated within a lipid moiety are also being developed and clinically tested as cancer vaccines. The lipids protect the RNA and mediate its endosomal uptake and release from the endosome. Further, the synthetic srRNA lacks a viral shell or any viral membrane proteins that could either induce, or be targeted by, neutralizing antibodies. This should allow repeated dosing and the ability to encode multiple and larger genes of interest, normally limited by the packaging capacity of the viral particle. Among the antigens that are being included in current cancer vaccines are both shared and neo-epitopes.

### Vaccine description

Vaccines based on neoepitopes derived from autologous tumor offer the possibility of a more potent, personalized immune response. In one individualized vaccine strategy referred to as GRANITE [16], neoantigens predicted by a proprietary model to fit within a patient’s HLA molecules are inserted into expression cassettes that are used to generate either a chimpanzee adenovirus (ChAd) (GRT-C901) or a self-amplifying mRNA (they refer to as SAM) formulated in lipid nanoparticles (GRT-C902). The adenovirus vaccine is used for a prime and the self-amplifying mRNA was used as a heterologous boost. A similar but off-the-shelf ChAd prime/SAM boost strategy called SLATE has also been developed in which the encoded antigens are shared neoantigens derived from common driver mutations (such as KRAS).

### Clinical study design

In the GRANITE phase I/II experience, patients with pretreated, advanced microsatellite stable colorectal cancer, gastroesophageal adenocarcinoma, and non-small cell lung cancer (previously treated with checkpoint inhibitors) were administered the heterologous prime/boost vaccine plus ipilimumab (30 mg SC) and nivolumab (480 mg IV). A second ChAd boost was also administered.

In the SLATE phase I/II, patients with pretreated non-small cell lung cancer, microsatellite stable colorectal cancer, pancreatic ductal adenocarcinoma, ovarian cancer, and ampullary adenocarcinoma with KRAS mutations were administered the off-the-shelf heterologous vaccines (encoding various KRAS mutations).

### Tolerability and toxicity

Among the 26 patients in GRANITE study, there were no DLTs, no vaccine discontinuations due to TRAEs, and the most common AEs were grade 1/2 fever (15 subjects) and injections site reactions (15 subjects). The majority of the grade 1/2 reactions and the rare grade 3/4 reactions were those reported with nivolumab or can occur with the underlying malignancies.

Among the SLATE treated patients, the adverse events were mainly grade 1/2 fever and injection site reactions.

### Immune responses

Prior to vaccination, most of the GRANITE patients had undetectable or minimally detectable neoantigen-specific T cell responses but after immunization, all analyzed patients had some increase in neoantigen-specific T cells ranging from a few IFNγ spot forming units (SFU)/10^6^ cells to >1500 SFU/10^6^ cells [17]. Vaccine-induced, neoantigen-specific T-cell responses that were activated by the prime dose were maintained or increased further by the self-amplifying RNA boosts. Elicited cytotoxic T-cells were specific for multiple neoantigens and infiltrated the tumor. Epitope spreading was also observed.

The SLATE immune response data has not been reported.

### Clinical response

Of 22 evaluable patients in the GRANITE study, there was 1 confirmed complete response lasting >4 months in a gastroesophageal cancer patient, and 5 stable disease (1 CRC > 18 months, 1 CRC > 9 months, 1 CRC and 1 GEA up to 6 months), 11 progressive disease (PD), and 2 no measurable disease. Of 9 pts treated beyond RECIST PD, 4 patients did not have confirmed PD at the next scan. In 9 pts with MSS-CRC with at least one scan, 5 were progression-free per iRECIST beyond 6 months and 4 of the 5 showed circulating tumor DNA (ctDNA) response (decrease ≥ 50% from baseline). Among these MSS CRC patients, molecular response (ctDNA reduction) was associated with an increased OS (>17 vs 7.8 months) and iPFS (11.8 vs 2 months) and PFS (4.9 vs 2 months).

In the SLATE study, one non-small cell lung cancer patient had an unconfirmed PR and 4 patients had SD. Among the NSCLC patients with KRAS G12C mutations (and HLA A*0101 type), ctDNA responses were observed in 66%.

## Conclusions

Clinical trials of srRNA vaccines based on viral replicon particles have demonstrated safety with minimal toxicity associated with the injections. Induction of antigen-specific T and B cell responses occur with adequate dose levels, despite the development of VRP–specific neutralizing antibodies. Fully synthetic srRNA delivered within lipid nanoparticles have been applied as part of prime-boost strategies and demonstrated potent boosting of immune responses. Lack of a viral shell reduces anti-vector immunity, allowing repeated dosing, and permits inclusion of multiple larger genes of interest within the expression cassette, otherwise limited by the packaging capacity of viral particles. Experience gained with srRNA and their delivery vehicles during their development for infectious disease indications will also aid the development of srRNA platforms for malignancy. For example, it will be important to determine whether reactogenicity to the components of the lipid nanoparticles (or impurities introduced during manufacturing) is greater than VRP. Further, the role and sequencing of coadministered immune checkpoint inhibitors will also need to be clarified for the various potential indications. In patients with advanced malignancies, combination with immune checkpoint blockade may be critical. We and others have observed srRNA vaccines in combination with immune checkpoint blockade result in increased T cell infiltration into tumor tissue. The ongoing randomized phase II study of VRP-HER2 plus pembrolizumab is designed to demonstrate whether the combination enhances intratumoral T cell responses and clinical activity in the setting of advanced disease.

## Data Availability

Not applicable.
